# Whole-genome sequence and phenotypic characteristics of *Bacillus cereus* strain IM8 isolated from the Upper Fremont Glacier in Wyoming

**DOI:** 10.1128/mra.01260-25

**Published:** 2026-03-16

**Authors:** Tyler Chandross-Cohen, Mira Ebersole, Nazeerah Rahman, Andrew S. Wagner, Jasna Kovac, Hans Wildschutte

**Affiliations:** 1 Department of Food Science, The Pennsylvania State University 730256, University Park, Pennsylvania, USA; 2 One Health Microbiome Center, The Pennsylvania State University 8082, University Park, Pennsylvania, USA; 3 Department of Biological Sciences, Bowling Green State University 110004, Bowling Green, Ohio, USA; Fluxus Inc., Sunnyvale, California, USA

**Keywords:** *Bacillus cereus*, glacier

## Abstract

*Bacillus cereus* strain IM8 isolated from an ice core of the Upper Fremont Glacier in Wyoming showed 97.52% average nucleotide identity with *B. cereus sensu stricto*, a lineage typically not associated with psychrotolerance. Phenotypic characterization highlighted high cytotoxicity toward human intestinal epithelial Caco-2 cells and resistance to ampicillin.

## ANNOUNCEMENT

Glacial environments serve as natural cryogenic libraries where microorganisms can remain preserved for multimillennial periods, providing opportunities to study them (1–3). The *Bacillus cereus* group comprises closely related, spore-forming gram-positive species of environmental, medical, and agricultural importance (4, 5). Here, we report the genome and phenotypic characterization of *B. cereus* IM8 isolated from the Fremont Glacier in Wyoming.

**Fig 1:**
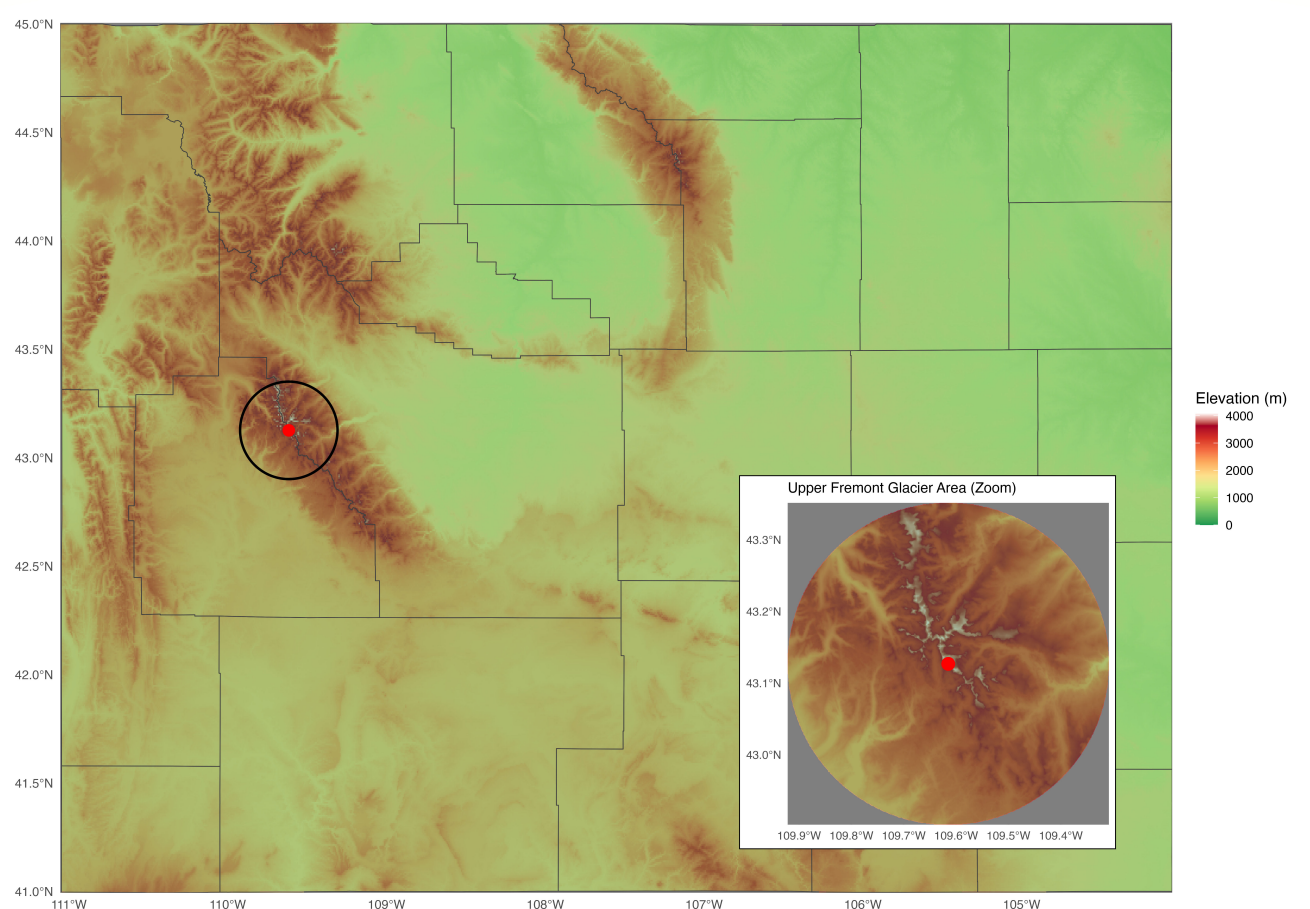
Terrain map and locator inset of the Upper Fremont Glacier sampling site in the Wind River Range, Wyoming, USA. The black circle denotes the zoomed inset. The inset panel shows a closer view of the glacier area surrounding the sampling location. The red pin indicates the precise ice core collection site (43°07′37″ N, 109°36′54″ W). Map created by the authors.

A 401.2 g ice core from the Upper Fremont Glacier, Wyoming (43°07′37″ N, 109°36′54″ W) was collected in 1991 at a 28 m depth using a sterile solar-powered drill (6) (Fig. 1) and stored at −20°C. On 8 April 2025, the core was melted aseptically, and 25 mL aliquots were filtered through 0.2 µm Whatman membranes (Millipore Sigma). Filters were plated on tryptic soy agar (TSA, BD) and incubated at 22°C for 48 h. Twenty-two colonies were isolated and preserved in 20% glycerol at −80°C. 16S rRNA genes were amplified as previously described (7) and Sanger sequenced at the University of Chicago Sequencing Center. National Center for Biotechnology Information (NCBI) BLAST analysis suggested that isolate IM8 was *B. cereus*, having 99.26% nucleotide identity, 100% query coverage, and an *E* value of 0 to *B. cereus* strain NPK1_1_39.

DNA extraction, WGS, and genome assembly were performed by SeqCenter (Pittsburgh, PA). An overnight culture streaked on TSA (BD) at 23°C was sent to SeqCenter. DNA extraction was performed with the ZymoBIOMICS DNA Miniprep Kit (Zymo Research), and DNA was quantified with Qubit 4 (Thermo Fisher Scientific). For Nanopore sequencing, libraries were prepared using 60 μL of DNA (40 ng/μL) with the Ligation Sequencing Kit (SQK-LSK114) and NEBNext Companion Module (E7180L) following the manufacturer’s instructions. No additional DNA fragmentation or size selection was performed. Nanopore sequencing was performed on a GridION using R10.4.1 flow cells, and Dorado (Version 0.5.3, super-accurate and 5mC/5hmC models) was used for basecalling and demultiplexing. For Illumina sequencing, libraries were prepared using 30 μL of DNA (10 ng/μL) with the Illumina DNA Prep Kit following the manufacturer’s instructions using custom 10 bp unique dual indices (UDI) and a target insert size of 280 bp. Illumina sequencing was performed on an Illumina NovaSeq X Plus sequencer, producing 2 × 151 bp paired-end reads. Demultiplexing, quality control, and adapter trimming were performed with bcl-convert1 (Version 4.2.4). Nanopore reads were assembled *de novo* using Flye (Version 2.9.2) (8). For hybrid assembly, Nanopore assembly genome polishing was performed with Pilon (Version 1.24) using the Illumina reads (9). The quality of the assembled genome was assessed using Quast (Galaxy Version 5.2.0) (10). BTyper3 (Version 3.4.0) (11) was used for taxonomic classification, and ABRicate (Galaxy version 1.0.1) (12) was used to detect antimicrobial resistance genes in the assembled genome. The genome was annotated using the NCBI Prokaryotic Genome Annotation Pipeline (Version 6.10). Default parameters were used for all analyses, except where otherwise noted.

Broth microdilution antibiotic susceptibility assays were performed in accordance with the Clinical Laboratory Standards Institute (CLSI) M45-A3 3rd edition guidelines (13). Cytotoxicity testing was performed as previously described (14) using Caco-2 cells (ATCC). Both antibiotic susceptibility assay and cytotoxicity results are found in Table 1.

**TABLE 1:** Genome sequencing statistics, genome properties, and antibiotic resistance profile

Genome Characteristics	Genome Statisctics
Number of Illumina reads (R1 + R2)	19,930,394
Total bp obtained for Illumina reads	2,856,171,373
Number of Nanopore reads	698,765
Total bp obtained for Nanopore reads	2,837,665,876
N50 of Nanopore reads	5,309
Number of contigs in assembly	3
Genome length (bp)	5,293,182
Number of CDSs^*a*^	5,259
Number of tRNAs^*a*^	106
Genome coverage	536.4×
GC content (%)	35.29
*panC* phylogenetic grouping and species (% ANI)^*b*^	Group IV, *B. cereus sensu stricto* (s.s.) (97.52%)
Closest type strain (% ANI)	*B. cereus sensu stricto* (s.s.)-type strain ATCC 14579 (98.03%)
Antibiotic resistance phenotype^*c*^	Resistant: AMP; sensitive: CHL, VAN, CIP, GEN
ABRicate-detected antimicrobial resistance genes^*d*^	*BcII and bla1* (β-lactam resistance, 100% identity), *fosB_gen* (fosfomycin resistance, 100% identity), *satA_Ba* (streptothricin acetyltransferase, 98.38% identity), *and vanZ-F* (putative vancomycin resistance-associated gene, 94.20% identity)
BTyper3-detected virulence genes	Non-hemolytic enterotoxin (*nheA*, *nheB*, *nheC*); hemolysin BL (*hblA*, *hblB*, *hblC*, *hblD*); cytotoxin K (*cytK-2*); sphingomyelinase (*sph*)
Average normalized cytotoxicity (± standard deviation)^*e*^	0.83 ± 0.07
SRA accession number	Illumina reads: SRR35898549, Nanopore reads: SRR35898549
GenBank accession number	JBRYQY000000000

^*a*^ Annotated using the NCBI Prokaryotic Genome Annotation Pipeline (Version 6.10) (15).

^*b*^ Determined using BTyper3 95.2% average nucleotide identity cutoff and phylogenetic grouping based on the *panC* gene sequence (11).

^*c*^ Five antimicrobials were tested in total: ampicillin (128–0.125 μg/mL; Fisher Scientific), chloramphenicol (32–0.3125 μg/mL; Fisher Scientific), vancomycin (16–0.015 μg/mL; Fisher Scientific), ciprofloxacin (4–0.003 μg/mL; Fisher Scientific), and gentamicin (128–0.25 μg/mL; Gibco). Minimum inhibitory concentrations (μg/mL) were used according to CSLI M45-A3 breakpoints. AMP, ampicillin (MIC > 128); CHL, chloramphenicol (MIC = 4); VAN, vancomycin (MIC = 1); CIP, ciprofloxacin (MIC = 0.125); and GEN, gentamicin (MIC = 0.5).

^*d*^ Detected using the NCBI bacterial antimicrobial resistance reference gene database (16).

^*e*^ A value of 0 indicates no cytotoxicity, and 1 indicates maximum cytotoxicity that equals that of the positive control (*B. cereus* ATCC 14579).

## Data Availability

The genome assembly and raw reads are available in NCBI under PRJNA1354427. The raw Illumina reads are available in NCBI in the Sequence Read Archive under the accession number SRR35898549, and the raw nanopore reads are available under the accession number SRR35898549. The assembled genome is available in GenBank under accession number JBRYQY000000000.
